# Evaluating diagnosis and treatment of oral and esophageal candidiasis in Ugandan AIDS patients.

**DOI:** 10.3201/eid0502.990214

**Published:** 1999

**Authors:** M Ravera, A Reggiori, A M Agliata, R P Rocco

**Affiliations:** Regional Teaching Hospital, Hoima, Uganda. petra@swiftuganda.com

## Abstract

A randomized cross-over clinical and endoscopic evaluation of 85 Ugandan patients showed that esophageal candidiasis in AIDS patients with oral candidiasis could be managed without endoscopy and biopsies. Oral lesions, especially when accompanied by esophageal symptoms, were sufficient for diagnosis. Miconazole was more effective than nystatin in treating esophageal candidiasis and could be a valid alternative to more expensive azolic drugs in developing countries.


### Evaluating Diagnosis and Treatment of Oral and Esophageal Candidiasis in Ugandan AIDS Patients

**Maurizio Ravera, Alberto Reggiori, Anna Maria Agliata, and Roberto Pidoto Rocco**
Regional Teaching Hospital, Hoima, Uganda

A randomized cross-over clinical and endoscopic evaluation of 85 Ugandan patients showed that esophageal candidiasis in AIDS patients with oral candidiasis could be managed without endoscopy and biopsies. Oral lesions, especially when accompanied by esophageal symptoms, were sufficient for diagnosis. Miconazole was more effective than nystatin in treating esophageal candidiasis and could be a valid alternative to more expensive azolic drugs in developing countries.

Candidiasis, a well-known opportunistic infection of AIDS patients, is the leading cause of infectious esophagitis (1,2). Studies show similar prevalence of *Candida* esophagitis in AIDS patients in the West (9.1% to 31%) (3-5) and in Africa (7.3% to 27%) (6-7). In most treated patients (80% to 100%) *Candida* esophagitis recurs after 3 months (8,9). Nevertheless, in patients with AIDS, candidiasis generally does not become systemic, and thus, clinical cure is important (9). Defining the most effective diagnostic and therapeutic approach to curing *Candida* esophagitis in AIDS patients is especially important in developing countries, which often have limited resources.

Diagnosis of esophageal candidiasis is usually based on the endoscopic appearance of the typical mucosal lesions and on histopathologic studies (10-12). Several western studies have shown that the diagnosis of this disease in AIDS patients can be made on clinical findings alone because the positive predictive value of esophageal symptoms as indexes of esophageal infection is 71% to 100% (10,13,14). Such an evaluation has not yet been made in African AIDS patients.

Several therapeutic regimens have been effective in treating oral and esophageal candidiasis (8,15-23). For the past decade, oral nystatin therapy has been considered effective in controlling *Candida* esophagitis (11). In tropical countries, the efficacy of nystatin in treating this disease is not well known, although a recent study in Zaire reported a cure rate of less than 10% (24). We evaluated the diagnostic accuracy of esophageal symptoms in predicting *Candida* esophagitis in Ugandan AIDS patients with oral candidiasis and compared the effectiveness of miconazole and nystatin in treating oral and endoscopically proven esophageal candidiasis in these patients.

### The Study

From September 1994 to December 1995, 320 consecutive AIDS patients were observed at the Gastroenterology Department of Hoima Hospital in Uganda. Among them, 85 (45 women, 40 men, mean age 27.1, standard deviation [SD] 5.3 years) fulfilled admission criteria: positive HIV test or clinical diagnosis of AIDS and presence of oral patchy white plaques as markers of oral candidiasis. The district medical officer and the hospital medical superintendent granted approval for the study, and informed consent was obtained from each patient. Patients were considered symptomatic if they had any of the following symptoms: odinophagia, dysphagia, or retrosternal burning pain.

All patients were hospitalized, and the upper digestive tract was examined endoscopically. The diagnosis of esophageal candidiasis was made at the examination. All patients had the

Address for correspondence: Maurizio Ravera, c/o AVSI P.O. Box 6785, Kampala, Uganda; fax: 256-41-266967; e-mail: petra@swiftuganda.com.

same spectrum of lesions: patchy white plaques, confluent pseudomembrane, and friable mucosa. Routine histopathologic assessment was not performed, mainly because of cost.

Patients were randomly assigned to the nystatin or miconazole regimen; a stratified randomization method was used to balance treatment groups by esophageal symptoms, age, and sex (Table). Nystatin tablets were given at a dose of 1,000,000 I.U. every 8 hours for 7 days (according to Uganda Ministry of Health 1993 National Standard Treatment Guidelines), while miconazole tablets were administered at a dose of 250 mg every 6 hours for 7 days (25). At a mean follow-up of 7.6 days (SD 0.9), the patients' symptoms were reassessed, and the upper digestive tract was reexamined endoscopically. The endoscopist was blind to the treatment used. Patients given nystatin who still had candidiasis were placed on the miconazole regimen and tested for candidiasis 1 week later.

Table. Characteristics of patients participating in the study

| | No. | Mean age (yr) (S.D.) | Sex M/F |
|---|---|---|---|
| Oral candidiasis | 85 | 24.0 (6.7) | 32 / 53 |
| Esophageal symptoms[a] | 40 | | 17 / 23 |
| Nystatin group | 20 | 23.9 (6.1) | 7 / 13 |
| Miconazole group | 20 | 23.7 (6.6) | 8 / 12 |
| Esophageal candidiasis | 77 | 23.7 (6.4) | 27 / 50 |
| Nystatin group | 37 | 24.2 (6.5) | 12 / 22 |
| Miconazole group | 40 | 23.4 (6.1) | 15 / 28 |

[a]Esophageal symptoms are any of the following: odinophagia, dysphagia, retrosternal burning pain.

### Findings

Most (90.8%) (42 female, 35 male, mean age 28.0 ± 5.8 years) of the study participants had both oral and esophageal candidiasis. Forty (47.1%) had esophageal symptoms, and all had esophageal candidiasis at endoscopy. Sensitivity, specificity, and the positive and negative predictive values of esophageal symptoms as markers of esophageal infection were 83.3% (confidence interval [CI] 69.2 to 92.0), 100% (CI 88.3 to 100), 100% (CI 89.1 to 100), and 82.2% (CI 67.4 to 91.5), respectively.

Esophageal symptoms disappeared in 10 (27.0%) of the 37 patients in the nystatin group and in 38 (95.0%) of 40 patients in the miconazole group (Yates chi-square = 34.99, p <0.001). Oral candidiasis was cured in all patients in both groups; esophageal candidiasis was cured in 8 (21.6%) patients in the nystatin group and in 37 (92.5%) patients in the miconazole group (Yates chi-square = 36.89, p <0.001). Of the 29 patients who did not respond to nystatin, 27 (93.1%) were cured with miconazole (Figure). No adverse effects were observed in either group.

More than 90% of AIDS patients with oral candidiasis in this study also had esophageal candidiasis, thus confirming that such an association is also very strong in Uganda (14,26). A little more than half (51.9%) of 77 patients with esophageal candidiasis also had esophageal symptoms. Our findings and those of other studies support the observation that esophageal candidiasis could be suspected if oral thrush is present, especially when esophageal symptoms are associated (10,14,26,27). Thus, in tropical countries, endoscopic assessment and biopsies might best be reserved for patients who have esophageal symptoms after receiving prolonged antifungal treatment to confirm diagnosis of candidiasis or to determine other infectious causes of this symptoms (e.g., herpes simplex virus infection, cytomegalovirus infection, cryptosporidiosis) (2,23).

Although our study was not designed to detect recurrence of candidiasis, esophageal candidiasis is likely to recur in AIDS patients within 12 months from any antifungal treat-

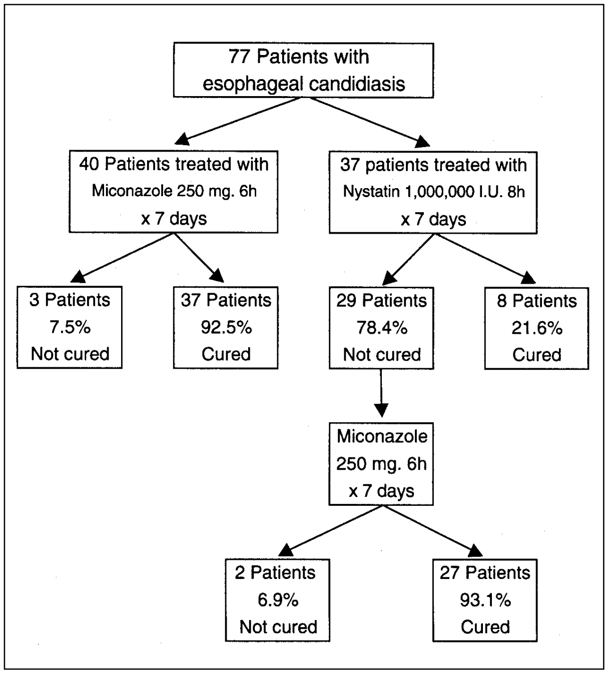

Figure. Esophageal candidiasis treatment flow.

ment; if it does, response to therapy is worse than response to initial therapy (28). For this reason, as well as the possibility of resistance to miconazole (sporadic cases have been reported [29]), more expensive azolic drugs (e.g., fluconazole) should be reserved for recurrences of the disease; the disease should be treated initially with less expensive, but more effective drugs (e.g., miconazole).

In sum, AIDS patients with oral candidiasis in countries similar to Uganda can be managed without endoscopic and bioptic assessments since oral lesions are typical and a high prevalence of esophageal involvement is expected (with or without symptoms) (>90% of cases in our study). In our patients, nystatin had a very low cure rate in the treatment of esophageal candidiasis in AIDS patients; however, it could still play a role in the treatment of oral candidiasis, especially in nonimmunocompromised patients, in whom concomitant esophageal involvement is less common. On the other hand, miconazole, a medium-priced azolic drug, was very effective and could be a valid alternative to more expensive azolic drugs in developing countries.

### Acknowledgments

We thank Dr. G. Oundo, medical superintendent, and the staff of Hoima Hospital.

This study was funded by International Service Volunteers' Association, Kampala, Uganda.

Dr. Ravera is a specialist in gastroenterology and digestive endoscopy. A researcher at the Italian National Health Institute, he serves as site coordinator/monitor of the UNAIDS PETRA study at Nsambya Hospital, Kampala, Uganda. His research interests include gastroenterology, endoscopy, infectious diseases, and HIV/AIDS.

### References

1. Wilcox CM, Karowe MW. Esophageal infections: etiology, diagnosis and management. Gastroenterologist 1994;2:188-206.
2. Lopez Dupla M, Mora Sanz P, Pintado Garcia V, Valencia Ortega E, Uriol PL, Khamashta MA, et al. Clinical, endoscopic, immunologic and therapeutic aspects of oropharyngeal and esophageal candidiasis in HIV infected patients: a survey of 114 cases. Am J Gastroenterol 1992;87:1771-6.
3. Sirera G, Clotet B, Romeu J. Incidence of opportunistic infections and malignancies in AIDS patients from Barcelona according to patients' risk behaviour. VII International Conference on AIDS, Florence Jun 16-21, 1991; M.B.2459 [abstract].
4. Heise W, Arasteh K, Mostertz P. Gastrointestinal manifestations in AIDS. Endoscopic, histological and microbiological aspects. VII International Conference on AIDS, Florence Jun 16-21, 1991:2227 [abstract].
5. Scevola D. Apparato gastrointestinale. In: Dianzani F, editor. Il libro italiano dell'AIDS. Milano: Mc Graw-Hill; 1994. p. 305-12.
6. Howlett WP, Nkya WM, Mmuni KA, Missalek WR. Neurological disorders in AIDS and HIV disease in the northern zone of Tanzania. AIDS 1989;3:289-96.
7. Colebunders R, Latif AS. Natural history and clinical presentation of HIV-1 infection in adults. AIDS 1991;5 Suppl 1:S103-12.
8. Smith DE, Midgley J, Allan M, Connolly GM, Gazzard B. Itraconazole versus ketoconazole in the treatment of oral and oesophageal candidiasis in patients infected with HIV. AIDS 1991;5:1367-71.
9. Laine L, Bonacini M. Esophageal disease in HIV infection. Arch Intern Med 1994;154:1577-82.
10. Connolly GM, Hawkins D, Harcourt-Webster JN, Parsons PA, Husain OA, Gazzard BG. Oesophageal symptoms, their causes, treatment and prognosis in patients with the acquired immunodeficiency syndrome. Gut 1989;30:1033-9.
11. Mathieson R, Dutta SK. Candida esophagitis. Dig Dis Sci 1983;28:365-70.
12. Kodsi BE, Wickremesinghe PC, Kozinn PJ, Iswara K, Golberg PK. Candida esophagitis: a prospective study of 27 cases. Gastroenterology 1976;71:715-9.
13. Bonacini M, Young T, Laine L. The causes of esophageal symptoms in human immunodeficiency virus infection. A prospective study of 110 patients. Arch Intern Med 1991;151:1567-72.
14. Bianchi Porro G, Parente F, Cernuschi M. The diagnosis of oesophageal candidiasis in patients with the acquired immune deficiency syndrome: is endoscopy always necessary? Am J Gastroenterol 1989;84:143-6.
15. Pons V, Greenspan D, Debruin M. Therapy for oropharyngeal candidiasis in HIV infected patients: a randomized, prospective multicenter study group. Journal of Acquired Immune Deficiency Syndrome 1993;6:1311-6.
16. Greenspan D. Treatment of oropharyngeal candidiasis in HIV positive patients. J Am Acad Dermatol 1994;31:S51-5.
17. Hernandez Sampelayo T. Fluconazole versus ketoconazole in the treatment of oropharyngeal candidiasis in HIV infected children multicenter study group. Eur J Clin Microbiol Infect Dis 1994;13:340-4.
18. Gil A, Lavilla P, Lopez Dupla M, Valencia E, Pintado V, Khamashta M, et al. Treatment of esophageal candidiasis with fluconazole in acquired immunodeficiency syndrome. Comparative study of 2 therapeutic schemes. Med Clin (Barc) 1992;98:612-7.
19. Berger TG. Treatment of bacterial, fungal and parasitic infections in the HIV infected host. Seminar in Dermatology 1993;12:296-300.
20. Sutton FM, Graham DY, Goodgame RW. Infectious esophagitis. Gastrointest Endosc Clin N Am 1994;4:713-29.
21. Soubry R, Clerinx J, Banyangiliki V. Comparison of itraconazole oral solution and fluconazole capsules in the treatment of oral and esophageal candidiasis in HIV infected patients. Preliminary results. VII International Conference on AIDS, Florence 16-21 Jun, 1991: M.B. 2201 [abstract].

22. De Wit S. Comparison of fluconazole and ketoconazole for oropharyngeal candidiasis in AIDS. Lancet 1989;1:746-8.
23. Laine L, Dretler RH, Conteas CN, Tuazon C, Koster FM, Sattler F, et al. Fluconazole compared with ketoconazole for the treatment of candida esophagitis in AIDS. A randomized trial. Ann Intern Med 1992;117:655-60.
24. Nyst MJ, Perriens JH, Kimputu L, Lumbila M, Nelson AM, Piot P. Gentian violet, ketoconazole and nystatin in oropharyngeal and esophageal candidiasis in Zairean AIDS patients. Annales de la Societé Belge de Médecine Tropicale 1992;72:45-52.
25. Grahame-Smith DG, Aronson JK. Oxford textbook of clinical pharmacology and drug therapy. 2nd ed. New York: Oxford University Press Inc.; 1992. p. 559.
26. Raufman JP, Rosenthal LE. Oral candidiasis as a marker for esophageal candidiasis in the acquired immunodeficiency syndrome. Ann Intern Med 1986;104:54-8.
27. Bassetti D, Canessa A. Micosi. In: Ferdinando Dianzani, editor. Il libro italiano dell'AIDS. Milano: Mc Graw-Hill; 1994. p. 224-8.
28. Parente F, Cerruschi M, Rizzardini G, Lazzarin A, Valsecchi L, Bianchi Porro G. Opportunistic infections of the esophagus not responding to oral systemic antifungals in patients with AIDS: their frequency and treatment. Am J Gastroenterol 1991;86:1729-34.
29. Holt RJ, Azmi A. Miconazole resistant candida. Lancet 1978;1:50-1.
